# The Gut Microbiome and Cardiovascular Disease

**DOI:** 10.7759/cureus.14519

**Published:** 2021-04-16

**Authors:** Andrea A Astudillo, Harvey N Mayrovitz

**Affiliations:** 1 Osteopathic Medicine, Nova Southeastern University, Dr. Kiran C. Patel College of Osteopathic Medicine, Davie, USA; 2 Medical Education, Nova Southeastern University, Dr. Kiran C. Patel College of Allopathic Medicine, Davie, USA

**Keywords:** gut microbiome, cardiovascular disease, gut flora, cardiac health, dysbiosis, atherosclerosis, microbiota, clostridium difficile, short chain fatty acids

## Abstract

Cardiovascular disease (CVD) is currently the leading cause of death worldwide. Although many well-known conditions cause CVD, recent research has suggested that alterations to the gut microbiome may also promote CVD. The gastrointestinal tract houses trillions of bacteria, some of which in large numbers are considered to be part of a healthy gut microbiome profile. These “good” bacteria have the ability to process and digest complex carbohydrates into short-chain fatty acids (SFCA). These SCFA serve as signaling molecules, immune-modulating molecules, and sources of energy. However, with gut dysbiosis, there is an overgrowth of certain bacteria and these bacteria overly produce phosphatidylcholine, choline, and carnitine into the waste product trimethylamine-N-oxide (TMAO). Elevated TMAO levels are associated with an increased risk of atherosclerosis, myocardial infarction, thrombosis, and stroke. Therefore, introducing therapeutic interventions that alter a dysbiotic gut profile back to a healthy gut microbiome may be the key to reducing the incidence of cardiovascular disease in some conditions. The purpose of this review is to critically examine and consolidate the relevant information bearing on this concept. Our goal is to provide the informational framework for the possible use of microbiome modification as an optional therapeutic modality.

## Introduction and background

Cardiovascular disease (CVD) is the leading global cause of death, accounting for 17.3 million deaths globally per year and one of every three deaths in the United States [1]. CVD is a term used to describe a variety of diseases and disorders that affect the blood vessels and ultimately the heart. Atherosclerosis, heart failure, and hypertension are a few among several conditions which can result in CVD [1-3]. Known risk factors leading to these conditions include smoking, obesity, and diabetes mellitus [4]. However, recent research suggests that gut dysbiosis, or abnormal changes to the gut microbiota flora, may also contribute extensively to the progression of CVD, byway of the production of metabolic products produced by pathogenic bacteria [5]. 

The GI tract is an organ system composed of the mouth, esophagus, stomach, and small and large intestines. Its primary function is to digest and absorb nutrients and macromolecules from the food ingested and expel the remainder as feces [6]. However, the importance of the flora that lives within the GI tract, known as the gut microbiome, is significant and complex. Throughout its development, the gut microbiome co-evolved with its host; the GI tract offered a stable environment, while the microbes provided a broad range of functions such as digestion of complex dietary macronutrients, production of nutrients and vitamins, defense against pathogens, and maintenance of the immune system [6]. With more than 100 trillion microbial cells and 100 different bacterial species comprising the human gut microbiome, it is no surprise that host homeostasis is greatly impacted by this essential and symbiotic relationship [7]. Although over 90% of bacteria in a healthy adult gut are of the Bacteroidetes and Firmicutes phyla, the bacteria that make up each human gut microbiome varies [8]. Environmental factors, such as diet and medication use, as well as genetic factors, all contribute to the type of bacteria that can colonize and thrive in the gut [9]. 

The formation of the gut microbiome begins at birth when massive bacterial colonization of the newborn occurs due to vaginal, fecal, and skin microbiota exposure [10]. The microbiota is further enhanced by the infant's nutrition. A majority of the bacteria found in the intestinal flora of breastfed infants are gram-positive Bifidobacterium, which are also found in human breast milk [11]. Predictably, changes in the growing infant's diet are accompanied by changes to their gut microbiome’s composition, ultimately impacting digestion, metabolism, and immunity. The purpose of this review is to critically examine and consolidate the relevant information bearing on this concept. Our goal is to provide the informational framework for the possible use of microbiome modification as an optional therapeutic modality.

Method

**Figure 1:**
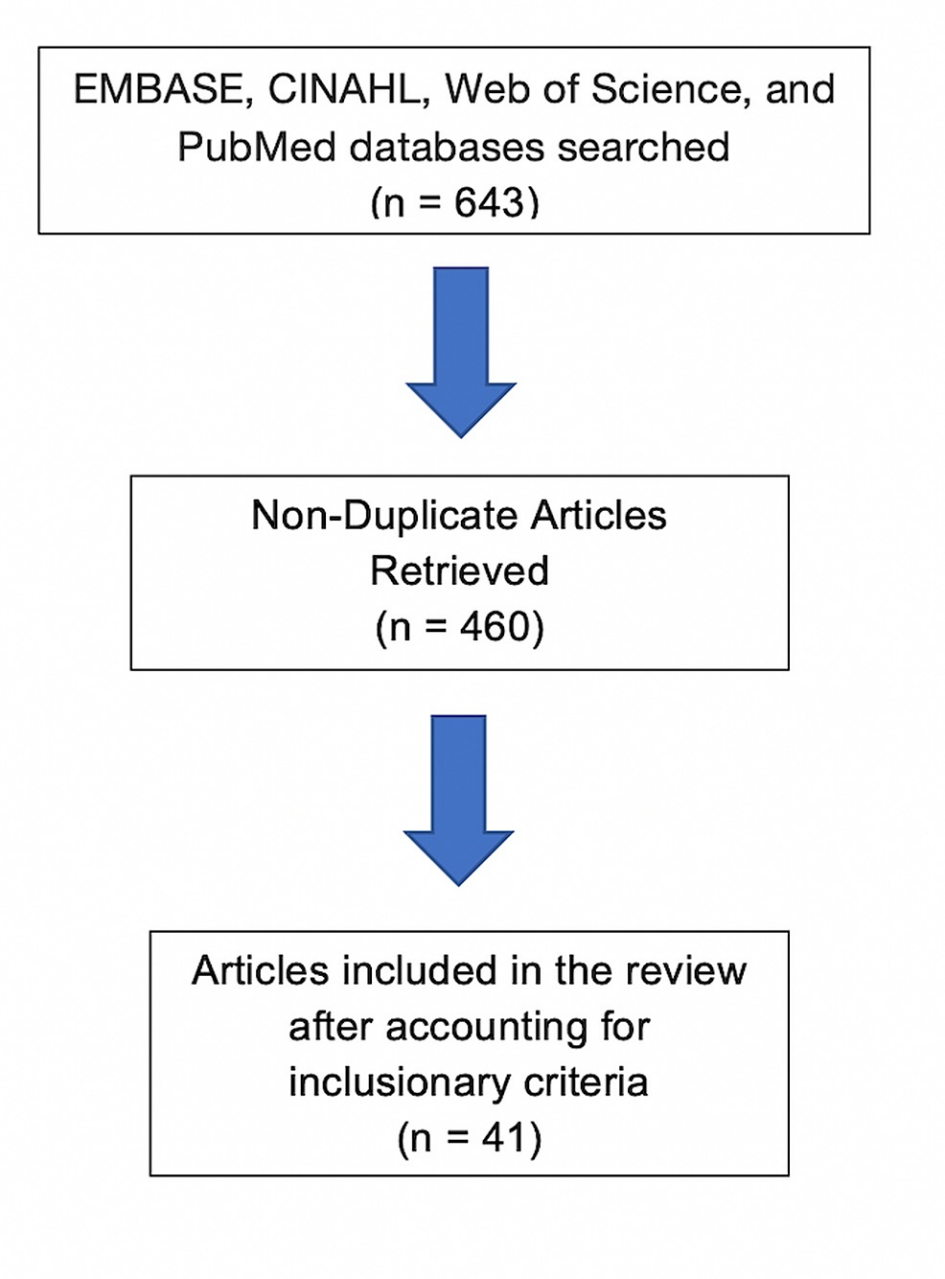
Search flow chart

The following databases were searched for articles in the English language: EMBASE, CINAHL, Web of Science, and PubMed. A total of 643 articles from these electronic databases were found after applying the search protocol which was the phrase “gut microbiome” in the title of the article and the phrase “cardiovascular disease” anywhere within the text. The search results yielded 201 articles from EMBASE, 71 from CINAHL, 175 from Web of Science, and 196 from PubMed. Out of these, 183 articles were found to be duplicated and removed yielding 460 articles. These were then screened for articles that emphasized gut microbiome and cardiovascular disease. Figure 1 shows a summary of the study selection process in a Preferred Reporting Items for Systematic Reviews and Meta-Analyses diagram.

## Review

The bacteria that comprise the gut microbiome create waste products that the body can utilize as molecules for metabolic and immune purposes. However, depending on the bacteria that metabolized them, these waste products can be beneficial or detrimental to homeostasis.

Metabolism and immunity

The gut microbiome ferments otherwise indigestible complex carbohydrates and proteins in the cecum and colon, producing short-chain fatty acids (SCFA), volatile fatty acids with fewer than six carbons [5]. The most abundant SCFA products of colonic bacterial fermentation are propionic acid, acetic acid, and butyric acid, representing 90-95% of the SCFA present in the colon [12]. These SCFA help to regulate host metabolic processes in order to achieve host homeostasis. When taken orally, propionic acid has been shown to increase satiety and improve glucose homeostasis [13]. Recent studies have also shown that butyric acid-producing Clostridiales strains (Roseburia and Faecalibacterium prausnitzii) were found to be decreased in patients with type 2 diabetes mellitus, but non-butyrate producing Clostridiales and pathogens such as Clostridium clostridioforme were increased [14,15]. Butyrate also prevented the development of insulin resistance and obesity, due to increased host energy expenditure [16].

SCFA are absorbed in the distal gut and are utilized by both the host and the microbiota as a source of energy. They also serve as signaling molecules that bind a variety of receptors to influence immune function and metabolism. G-protein coupled receptors (GPCR) such as GPCR41 and GPCR43 are expressed in adipose tissue, intestines, and immune cells [17]. By binding GPCR43, an essential receptor in neutrophil recruitment, SCFA help in regulating the inflammatory response [18]. SCFA are also able to interact with free fatty acid receptors (FFA) FFA2 and FFA3, influencing the expression of peptides and hormones which affects host energy and appetite regulation [19]. The gut microbiota also affects host metabolism by altering the composition of bile acids. Primary bile acids, which have escaped reabsorption, are converted into secondary bile acids by colonic bacteria. A small amount of these secondary bile acids enter circulation and act as hormones, influencing signaling pathways involved in energy expenditure, metabolism, and inflammation [20].

The gut microbiome also serves as a key factor in immune system modulation and maintenance of the gut mucosal intestinal barrier. Intestinal epithelial cells detect bacteria and other microbes through toll-like receptors (TLRs) and other pattern recognition receptors (PRR). Activation of PRR leads to both the activation of an immune response as well as a pathway to sustain the gut barrier, preventing systemic translocation of bacteria and protecting the host from infection [21]. The size and function of the regulatory T cell network are regulated by butyrate, which works to promote the induction and fitness of regulatory T cells in the colonic environment [22]. It is evident that gastrointestinal health relies heavily upon a well-functioning gut microbiome. 

Gut dysbiosis and CVD

Gut dysbiosis occurs when the gut microbiome is significantly altered due to a change in environmental factors, allowing opportunistic pathogens to colonize the gut in place of the healthy bacteria that typically thrive in optimal conditions. Changes in diet, antibiotic use, and new-onset or progression of disease are factors that alter the gastrointestinal environment. As previously stated, the metabolic products of a healthy gut microbiome regulate host immunity and metabolism, functioning as signaling molecules for several receptors to maintain host homeostasis. However, in a state of dysbiosis, the new colonic bacteria fail to produce the required metabolic products necessary for optimal function, leading to disruption in homeostasis. Additionally, other metabolic products may be produced which can harm the host. This chain reaction leads to immune and metabolic dysfunction, rendering the host vulnerable to disease. 

**Figure 2:**
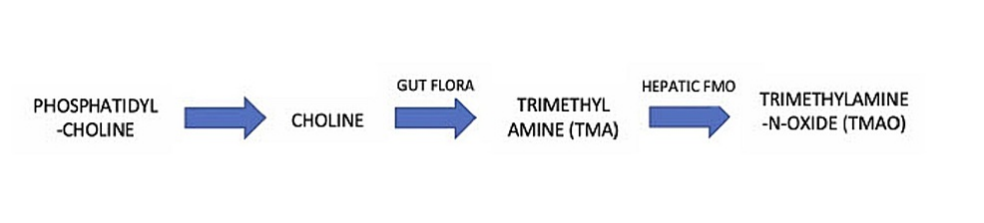
TMAO production process Flavin monooxygenase (FMO)

Trimethylamine-N-oxide (TMAO), a small-molecule metabolite, has recently been believed to serve as a link between the gut microbiota and host progression to CVD. Trimethylamine (TMA) is the waste product produced after TMA-containing nutrients, such as phosphatidylcholine, choline, and carnitine, fermented by gut microbes as a carbon fuel source [23,24]. TMA is taken to the liver via the portal circulation and is converted into TMAO by a host enzyme, hepatic flavin monooxygenase (FMO) [23]. Foods rich in choline include eggs, milk, liver, red meat, and poultry, and are therefore sources of TMA with which the host can produce TMAO [25]. Figure 2 illustrates the process by which the gut flora metabolizes phosphatidyl-choline into TMAO metabolites. 

Multiple independent cohorts have been able to relate the rising TMAO levels to adverse clinical outcomes. In a human study, more than 1800 patients undergoing elective coronary angiography showed that all TMA metabolites, including choline, had a positive association with prevalent CVD and incident cardiovascular events [23]. In a subsequent study of over 4000 patients undergoing elective coronary angiography, elevated TMAO levels were associated with an increased risk of death, myocardial infarction, and stroke over a three-year follow-up period [26]. Patients in the highest quartile of plasma TMAO levels were shown to have a 2.5-fold higher risk of a major adverse cardiovascular event than did patients in the lowest quartile [26]. Furthermore, elevated TMAO in fasting plasma was found to be a predictor of major adverse cardiovascular events independent of traditional cardiovascular risk factors and the presence or extent of coronary artery disease [5]. Elevated TMAO levels also lead to the increased macrophage cell formation and atherosclerotic development, as shown in animal model studies fed a dietary supplement of choline [23]. Atherosclerosis leads to the narrowing of blood vessels, which can result in a myocardial infarction or stroke. 

Conversely, suppression of intestinal microbiota by the broad-spectrum antibiotics essentially eliminates TMAO production and subsequent atherosclerosis development [26]. In an animal study where mice were fed a choline diet, half were treated with the broad-spectrum antibiotics to suppress intestinal microflora and the other half remained with intact microflora. Observed in antibiotic-treated mice was the complete inhibition of dietary choline-mediated enhancement in atherosclerosis [23]. However, choline supplementation in mice with intact intestinal microbiomes augmented atherosclerosis nearly three-fold. Intact gut microbiota is therefore seemingly essential to enhancing CVD risk effects of choline metabolism. 

In addition to increasing atherosclerotic activity, TMAO has been shown to intensify platelet hyperreactivity and thrombosis risk. In an in vivo experiment, exposure of platelet-rich plasma from healthy human volunteers (N>4000) to TMAO demonstrated the enhanced platelet activation from multiple agonists through increased Ca2+ release from intracellular stores [27]. In this human cohort, there was a dose-dependent association between plasma levels of TMAO and incident risk for thrombotic events [27]. In a five-year study, TMAO levels were associated with higher all-cause mortality among 821 patients with adjudicated peripheral artery disease [28]. These observations suggest that TMAO could be a marker for coronary plaque progression and can be assessed as a direct participant in an enhanced risk for CVD.

Risk factors

Established risk factors for CVD include hypertension, heart failure, and diabetes mellitus - all of which are believed to be associated with dysbiosis of the gut. Recent studies found elevated blood pressures to be prevalent in germ-free rats, suggesting that the gut microbiota is necessary for proper blood pressure regulation [29]. Additionally, when the fecal microbiota of spontaneously hypertensive rats and chronic angiotensin II infusion rat models of hypertension were compared, significant dysbiosis was noted [30]. This was found to be due to the decreased microbial richness, diversity, and increased Firmicutes/Bacteroidetes ratio in the hypertensive animals [30].

SCFA are also found to affect blood pressure. A product of gut microbial metabolism, SCFA can stimulate GPCR and therefore have a broad impact on host physiology, including pathways that impact renin secretion and blood pressure regulation. For example, in a series of studies done using renal and vascular olfactory receptor (Olfr) 78 and GPR41 knockout mice, blood pressure was shown to be altered [31]. Stimulation of Olfr78 was observed to elevate blood pressure, whereas stimulation of GPR41 lowered the blood pressure [31]. Additionally, the butyrate-producing genus Odoribacter was associated with lower blood pressure in overweight and obese pregnant women [32]. These data suggest that there is a link between gut microbiota metabolic products and alteration of blood pressure. 

Heart failure, another major risk factor for CVD, has also been shown to be affected by the state of the gut microbiome. In the "gut hypothesis of heart failure," it is believed that the decreased cardiac output and elevated systemic congestion can cause intestinal mucosal ischemia and edema, therefore contributing to underlying inflammation by increasing bacterial translocation and circulating endotoxins [33]. In a study, heart failure patients with decreased intestinal blood flow were shown to have higher serum concentrations of immunoglobulin A-anti polysaccharide. This was correlated with an increase in bacterial growth, as determined from biopsies of colonic mucosa [34]. The type of bacterial flora in these subjects differed from that in control subjects, suggesting that the type of bacterial species that thrive in the gut can lead to heart failure progression [34]. TMAO levels have also been linked to heart failure development. Circulating TMAO levels were found to be higher in patients with heart failure as compared to those patients of the same age and gender without heart failure [35]. Recent animal model studies suggest that the TMAO pathway may directly contribute to the development of adverse ventricular remodeling and heart failure phenotype [32]. For example, mice fed a high choline diet had both higher TMAO levels and accelerated adverse ventricular remodeling compared to mice fed a sufficient but low choline diet [36]. An increase in fibrosis was also observed in mice on the high choline diet [36]. Although more concrete data needs to be produced in order to definitively link an increase in TMAO/choline to the progression of heart failure, this area seems to be worthy of further investigation. 

Other unsuspecting factors which affect the gut microbiome may also indirectly lead to CVD. For example, patients who suffer from bowel diseases may have a higher incidence of cardiovascular disease due to an abnormal GI microbiome. Patients with inflammatory bowel disease (IBD), a chronic intestinal condition, have an up to three-fold higher risk for developing venous thromboembolic (VTE) complications compared to the general population [37]. Additionally, mucin degrading bacterial species such as Lachnospiraceae and Ruminococcus are more abundant in patients with irritable bowel syndrome (IBS) [38]. As previously mentioned, damage to the intestinal barrier can lead to systemic bacterial translocation, leading to circulating endotoxins and ultimately inflammation. 

Therapeutic intervention

Alterations in the diet have proved to be an effective strategy in reducing cardiovascular risk and maybe an area with potential therapeutic benefits. Although the gut microbiome remains a relatively stable entity throughout the course of an individual’s lifetime, microbiota composition can rapidly change due to dietary interventions [39]. For example, fiber-rich diets were shown to promote the growth of beneficial commensal bacteria and limit the growth of known opportunistic bacteria [40]. Additionally, consumption of a high-fiber diet led to an increase in acetate-producing microbiota, lower blood pressure, and a decrease in cardiac hypertrophy and fibrosis [41]. Elimination of animal meats may also be beneficial for decreasing CVD risk. When comparing intestinal microbiota composition and function between omnivores and vegans/vegetarians, the results showed major differences in the gut microbial capacity to produce TMA and TMAO from dietary l-carnitine, with vegetarians and vegans having the minimal capacity to form TMA from carnitine [42]. The Western diet, a known risk factor for CVD, is characterized by high intakes of red and processed meats, high-fat dairy products, fried foods, and refined grains. 

In a study where mice were fed a Western diet, results showed greater plasma TMAO concentrations and the development of cardiac dysfunction and heart fibrosis [43] Additionally, scientists observed elevated expression of tumor necrosis factor-α (TNF-α) and interleukin-1β (IL-1β) and a decrease in the expression of anti-inflammatory cytokines (IL-10) [43]. In contrast, the Mediterranean diet (MD) consists of an array of minimally processed plant-based food items, such as vegetables, fruit legumes, olive oil, and seafood. In a study of 153 volunteers assessed over four cities in Italy, it was found that consumption of MD acceptable fruits, vegetables, and legumes led to an increase in fecal SFCA levels [44]. This can be attributed to fermentation by more bacteria belonging to Firmicutes and Bacteroidetes [44]. It was also found that lower adherence to the MD resulted in elevated levels urinary TMAO levels [44]. A diet chockfull of minimally processed, plant-based items is seemingly essential for gut microbial maintenance and health and can lead to decreased levels of TMAO, thereby lowering cardiovascular disease risk. The patient’s diet is an important facet of health that can be targeted in order to prevent the advancement of, and possibly reverse disease processes.

Fecal microbiota transplant (FMT) is a potential therapeutic tool that has been shown to treat intestinal illnesses. FMT is an effective treatment for patients infected with antibiotic-resistant Clostridium difficile and has been shown to induce an 80% remission rate [45]. Fecal transplants from lean healthy donors were transferred to overweight patients with metabolic syndrome, which after six weeks showed improved hepatic and peripheral insulin sensitivity [46]. The fecal transfer also led to an increase in gut microbial richness, specifically increasing butyrate-producing bacteria [46]. Fecal microbiota transplants from lean donors to insulin-resistant individuals with metabolic syndrome have been shown to increase insulin sensitivity and the number of microbiota producing butyrate, an SCFA known to affect satiety hormones [46]. This protocol could possibly help patients with an elevated pathogenic gut microbiome profile, attain healthy levels of SCFA producing bacteria. Additionally, personalized nutrition therapy can be utilized for diet-based interventions.

## Conclusions

Growing evidence has shed light on the significance of the gut microbiome and its influence on host physiology. SCFA, the fermented products of healthy gut bacteria, serve as signaling molecules and play a major role in immune function, metabolism, and maintenance of the gut mucosal intestinal barrier, all important processes that are required for homeostasis. However, human and animal model studies have elucidated that the presence of pathogenic gut microbiome composition can lead to elevated levels of TMAO metabolites, thereby progressing to hypertension, heart failure, and diabetes mellitus, and ultimately resulting in CVD. It is therefore imperative that the proper gut microbiome profile be not only attained but maintained, in an attempt to procure optimal health. Proposed interventions to cultivate a healthy gut microbiome include fiber-rich diets as well as the well-known Mediterranean diet, both of which promote the growth of beneficial commensal bacteria and limit the growth of known opportunistic bacteria. In the future, personalized therapeutic interventions such as personalized diets and fecal microbiota transplants will likely be utilized to target an individual’s gut microbiome and ensure adequate gut microbial composition. Further research on the effect of gut microbiota profile and its products on the levels of cytokines and biomarkers would be useful. This could provide information on an individual’s inflammatory state and disease progression or remission.

## References

[1] Sacks FM, Lichtenstein AH, Wu JHY (2017). Dietary fats and cardiovascular disease: a presidential advisory from the American Heart Association. Circulation.

[2] Hollander W (1976). Role of hypertension in atherosclerosis and cardiovascular disease. Am J Cardiol.

[3] Best PJ, Lerman A (2000). Endothelin in cardiovascular disease: from atherosclerosis to heart failure. J Cardiovasc Pharmacol.

[4] Balakumar P, Maung-U K, Jagadeesh G (2016). Prevalence and prevention of cardiovascular disease and diabetes mellitus. Pharmacol Res.

[5] Ahmadmehrabi S, Tang WHW (2017). Gut microbiome and its role in cardiovascular diseases. Curr Opin Cardiol.

[6] Koh A, De Vadder F, Kovatcheva-Datchary P, Bäckhed F (2016). From dietary fiber to host physiology: short-chain fatty acids as key bacterial metabolites. Cell.

[7] Qin J, Li R, Raes J (2010). A human gut microbial gene catalogue established by metagenomic sequencing. Nature.

[8] Rinninella E, Raoul P, Cintoni M, Franceschi F, Miggiano GAD, Gasbarrini A, Mele MC (2019). What is the healthy gut microbiota composition? A changing ecosystem across age, environment, diet, and diseases. Microorganisms.

[9] Pickard JM, Zeng MY, Caruso R, Núñez G (2017). Gut microbiota: role in pathogen colonization, immune responses, and inflammatory disease. Immunol Rev.

[10] Mueller NT, Bakacs E, Combellick J, Grigoryan Z, Dominguez-Bello MG (2015). The infant microbiome development: mom matters. Trends Mol Med.

[11] Mikami K, Kimura M, Takahashi H (2012). Influence of maternal bifidobacteria on the development of gut bifidobacteria in infants. Pharmaceuticals (Basel).

[12] Ríos-Covián D, Ruas-Madiedo P, Margolles A, Gueimonde M, de Los Reyes-Gavilán CG, Salazar N (2016). Intestinal short chain fatty acids and their link with diet and human health. Front Microbiol.

[13] Arora T, Sharma R, Frost G (2011). Propionate. Anti-obesity and satiety enhancing factor?. Appetite.

[14] Qin J, Li Y, Cai Z (2012). A metagenome-wide association study of gut microbiota in type 2 diabetes. Nature.

[15] Udayappan SD, Hartstra AV, Dallinga-Thie GM, Nieuwdorp M (2014). Intestinal microbiota and faecal transplantation as treatment modality for insulin resistance and type 2 diabetes mellitus. Clin Exp Immunol.

[16] Gao Z, Yin J, Zhang J (2009). Butyrate improves insulin sensitivity and increases energy expenditure in mice. Diabetes.

[17] Kimura I, Ozawa K, Inoue D (2013). The gut microbiota suppresses insulin-mediated fat accumulation via the short-chain fatty acid receptor GPR43. Nat Commun.

[18] Sina C, Gavrilova O, Förster M (2009). G protein-coupled receptor 43 is essential for neutrophil recruitment during intestinal inflammation. J Immunol.

[19] Sleeth ML, Thompson EL, Ford HE, Zac-Varghese SE, Frost G (2010). Free fatty acid receptor 2 and nutrient sensing: a proposed role for fibre, fermentable carbohydrates and short-chain fatty acids in appetite regulation. Nutr Res Rev.

[20] Chiang JY (2013). Bile acid metabolism and signaling. Compr Physiol.

[21] Cerf-Bensussan N, Gaboriau-Routhiau V (2010). The immune system and the gut microbiota: friends or foes?. Nat Rev Immunol.

[22] Belkaid Y, Hand TW (2014). Role of the microbiota in immunity and inflammation. Cell.

[23] Wang Z, Klipfell E, Bennett BJ (2011). Gut flora metabolism of phosphatidylcholine promotes cardiovascular disease. Nature.

[24] Bennett BJ, de Aguiar Vallim TQ, Wang Z (2013). Trimethylamine-N-oxide, a metabolite associated with atherosclerosis, exhibits complex genetic and dietary regulation. Cell Metab.

[25] Zeisel SH, Mar MH, Howe JC, Holden JM (2003). Concentrations of choline-containing compounds and betaine in common foods. J Nutr.

[26] Tang WH, Wang Z, Levison BS (2013). Intestinal microbial metabolism of phosphatidylcholine and cardiovascular risk. N Engl J Med.

[27] Zhu W, Gregory JC, Org E (2016). Gut microbial metabolite TMAO enhances platelet hyperreactivity and thrombosis risk. Cell.

[28] Senthong V, Wang Z, Fan Y, Wu Y, Hazen SL, Tang WH (2016). Trimethylamine N-oxide and mortality risk in patients with peripheral artery disease. J Am Heart Assoc.

[29] Honour J (1982). The possible involvement of intestinal bacteria in steroidal hypertension. Endocrinology.

[30] Yang T, Santisteban MM, Rodriguez V (2015). Gut dysbiosis is linked to hypertension. Hypertension.

[31] Pluznick JL, Protzko RJ, Gevorgyan H (2013). Olfactory receptor responding to gut microbiota-derived signals plays a role in renin secretion and blood pressure regulation. Proc Natl Acad Sci U S A.

[32] Gomez-Arango LF, Barrett HL, McIntyre HD, Callaway LK, Morrison M, Dekker Nitert M (2016). Increased systolic and diastolic blood pressure is associated with altered gut microbiota composition and butyrate production in early pregnancy. Hypertension.

[33] Sandek A, Bauditz J, Swidsinski A (2007). Altered intestinal function in patients with chronic heart failure. J Am Coll Cardiol.

[34] Sandek A, Swidsinski A, Schroedl W (2014). Intestinal blood flow in patients with chronic heart failure: a link with bacterial growth, gastrointestinal symptoms, and cachexia. J Am Coll Cardiol.

[35] Tang WH, Wang Z, Fan Y (2014). Prognostic value of elevated levels of intestinal microbe-generated metabolite trimethylamine-N-oxide in patients with heart failure: refining the gut hypothesis. J Am Coll Cardiol.

[36] Organ CL, Otsuka H, Bhushan S (2016). Choline diet and its gut microbe-derived metabolite, trimethylamine N-oxide, exacerbate pressure overload-induced heart failure. Circ Heart Fail.

[37] Zezos P, Kouklakis G, Saibil F (2014). Inflammatory bowel disease and thromboembolism. World J Gastroenterol.

[38] Jeffery IB, Das A, O'Herlihy E (2020). Differences in fecal microbiomes and metabolomes of people with vs without irritable bowel syndrome and bile acid malabsorption. Gastroenterology.

[39] David LA, Maurice CF, Carmody RN (2014). Diet rapidly and reproducibly alters the human gut microbiome. Nature.

[40] Foye OT, Huang IF, Chiou CC, Walker WA, Shi HN (2012). Early administration of probiotic Lactobacillus acidophilus and/or prebiotic inulin attenuates pathogen-mediated intestinal inflammation and Smad 7 cell signaling. FEMS Immunol Med Microbiol.

[41] Marques FZ, Nelson E, Chu PY (2017). High-fiber diet and acetate supplementation change the gut microbiota and prevent the development of hypertension and heart failure in hypertensive mice. Circulation.

[42] Koeth RA, Wang Z, Levison BS (2013). Intestinal microbiota metabolism of L-carnitine, a nutrient in red meat, promotes atherosclerosis. Nat Med.

[43] Chen K, Zheng X, Feng M, Li D, Zhang H (2017). Gut microbiota-dependent metabolite trimethylamine N-oxide contributes to cardiac dysfunction in Western diet-induced obese mice. Front Physiol.

[44] De Filippis F, Pellegrini N, Vannini L (2016). High-level adherence to a Mediterranean diet beneficially impacts the gut microbiota and associated metabolome. Gut.

[45] Brandt LJ, Aroniadis OC, Mellow M (2012). Long-term follow-up of colonoscopic fecal microbiota transplant for recurrent Clostridium difficile infection. Am J Gastroenterol.

[46] Vrieze A, Van Nood E, Holleman F (2012). Transfer of intestinal microbiota from lean donors increases insulin sensitivity in individuals with metabolic syndrome. Gastroenterology.

